# Development of a new decellularization protocol for the whole porcine heart

**Published:** 2021-08-08

**Authors:** Ana Lídia Jacintho Delgado, Ana Claudia Oliveira Carreira, Hianka Jasmyne Costa de Carvalho, Renata Kelly da Palma, Taís Harumi de Castro Sasahara, Carla Maria Figueiredo de Carvalho, Marisol León, Rodrigo da Silva Nunes Barreto, Maria Angélica Miglino

**Affiliations:** Department of Surgery, School of Veterinary Medicine and Animal Science, University of São Paulo, São Paulo, Brazil

**Keywords:** scaffold, protocol, porcine, heart, tissue engineering

## Abstract

**Background:**

Cardiovascular diseases are the leading cause of death in many countries. Advances in technology have been promoted in this regard, especially in tissue engineering, to meet the need for tissue or organ grafts. In this way, the porcine model has been used due to its morphophysiological similarity between the human species, mainly regarding the cardiovascular system. Tissue engineering is employed using biological scaffolds that are currently derived from porcine. These scaffolds are produced by decellularization, a process to remove cells aiming to maintain only its three-dimensional structure, formed by extracellular matrix (ECM). Its main objective is to produce organs through recellularized scaffolds that could eventually substitute the ones with impaired functions.

**Aim:**

In this way, the present study aimed to establish a new protocol for porcine heart decellularization with potential application on tissue engineering.

**Methods:**

A porcine heart aorta was cannulated with a silicon tube, and the organ was washed in 0.1% phosphate-buffered saline through a peristaltic pump (Harvard Peristaltic Pump – Harvard Apparatus). After that, deionized water was introduced in the same system. The decellularization procedure was carried out using ionic and non-ionic detergents, namely 4% sodium dodecyl sulfate (SDS) and 1% Triton X-100, respectively. SDS was perfused through myocardial circulation at 400 mL/min for 24 h for 6 days. Subsequently, the heart was infused with Triton X-100 and washed by PBS and water for 24 h. The heart volume was measured before and after the recellularization. After macroscopic evaluation, the heart samples were processed and stained by Hematoxylin and Eosin, Masson’s Trichrome, Weigert-Van Gieson, Alcian Blue, and Pricrosirius Red techniques for microscopic analysis. To observe the cell adhesion, the recellularization was provided in this scaffold, which was analyzed under immunofluorescence and scanning electronic microscopy.

**Results:**

The protocol provided cells remotion, with adequate concentration of remaining DNA. ECM components as collagen type I, elastin, and glycosaminoglycans were successfully maintained. The scaffold showed a high cells adherence and proliferation in the recellularization process.

**Conclusion:**

According to results, the protocol described in this work preserved the ECM components and the organ architecture, minimizing ECM loss and being possible to state that it is a promising approach to tissue bioengineering.

**Relevance for Patients:**

This study provides a protocol for whole porcine heart decellularization, which will ultimately contribute to heart bioengineering and may support further studies on biocompatibility relationship of new cells with recellularized scaffolds.

## 1. Introduction

Cardiovascular diseases are responsible for an elevated number of deaths in the world. The scarcity of donors allied with the wide number of people who need a heart transplant or heart graft makes tissue and organ bioengineering important agents on this current scenario [1]. In the medical bioengineering, it is possible to appoint the decellularization process, a promising technique that promotes cell removal while preserving the native extracellular matrix (ECM) [2-4].

The ECM preservation in the bioscaffolds obtained can act as an ideal environment for sustenance, proliferation, and distribution of new cells serving as mechanical support, and its use can be applied in regenerative medicine to increase the bioavailability of tissues and organs, reducing rejection after transplants [5]. When considering viability of products generated for grafts or transplants from this technique, even when they came from xenogeneic origins, the tissue engineering can become of remarkable concern [1,6].

As example, porcine heart scaffolds have been considered as an alternative production method of xenogeneic tissue and organs for heart transplantation in humans, due to the similar morphophysiological characteristics between the porcine cardiovascular system and the human cardiovascular systems, especially when considering its hemodynamics [7]. The decellularization method is essential in the manufacture of bioscaffolds, and it can be provided by physical and chemical methods, or both. The chosen detergent, its concentration, and processing time should be adjusted according to demand while considering the success of the procedure [5].

Studies have been developed protocols for decellularization in porcine heart valves, myocardium, and pericardium [4,8-10]. However, few protocols are established for decellularization of the entire porcine heart [11,12]. Thus, this study aimed to produce a new, efficient, practical, and low-cost decellularization protocol for whole porcine heart, prioritizing the preservation of the ECM proteins and proving its capacity for cell adhesion.

## 2. Materials and Methods

### 2.1. Material and reagents

This study was authorized by the Animal Experimentation Ethics Committee (1166080618). Adult porcine hearts of both sexes were obtained under clean conditions from a slaughterhouse. Each heart weighing approximately 300 g, was submitted to decellularization protocol based on the previously literature [4,8-10,13,14]. Protocols were tested, modified, and evaluated macroscopically and microscopically and the best one is described and detailed in this work.

For heart decellularization, connectors were purchased from peristaltic pump (Harvard Peristaltic Pump – Harvard Apparatus). About 4% sodium dodecyl sulfate (SDS) solution was prepared (Affymetrix, Santa Clara, CA). Hypertonic and hypotonic solutions were made from sodium chloride (Affymetrix). The phosphate-buffered saline (PBS) was prepared with sodium chloride (Affymetrix), sodium phosphate (Sigma-Aldrich, St. Louis, MO), potassium chloride (Sigma-Aldrich), and potassium phosphate (Sigma-Aldrich).

### 2.2. Preparation of porcine heart for decellularization and decellularization protocol

For detergents perfusion, the aorta was cannulated with a silicon tube. Then, the heart was placed in a beaker containing 4 L of 0.1% PBS solution, in a circulating system of 400 mL/min for 30 min, performed by a peristaltic pump (Harvard Peristaltic Pump – Harvard Apparatus). After this procedure, deionized water was perfused in the same system. Four liters of 4% SDS were perfused through myocardial circulation, at 400 mL/min for 24 h. This process was repeated for 6 days, and then, 1% Triton X-100 was infused, followed by PBS 0.1% solution and deionized water for 24 h to remove cell debris and chemical residues in the sample. This heart was submitted to macroscopic and microscopic analysis.

A non-decelullarized (native) porcine heart was used in the analysis as a control sample (native tissue) in the comparison to the decellularized organ, highlighting the cells removal and preservation of ECM.

### 2.3. Macroscopic evaluation

Macroscopic evaluation of native and decellularized heart was provided according to previously described in literature, taken account the color aspect, size, and the organ format.

### 2.4. Histological evaluation

For an accurate analysis, the technique of Systematic Uniform Random Sampling (SURS) collection [15] was applied in the histological processing. The native and decellularized hearts were dissected and random samples cut into 5–6 pieces from transverse portions perpendicular to the apex-base axis with 2 cm thickness. All fragments included the left ventricular area, which were selected for the histological characterization.

The left ventricle (LV) fragments were frozen and included in OCT (#4583-1, Sakura-Torrance, USA), sectioned with a cryostat (Leica) followed by the staining of these sections with hematoxylin-eosin (HE), Masson’s Trichrome, Weigert-Van Gieson, Alcian Blue, and Picrosirius red techniques to assess the presence of nuclei and ECM structures. Slides stained by Picrosirius Red were also observed under a polarized microscope. A qualitative description of the images obtained from these slides was performed.

### 2.5. Quantification of remaining genomic DNA

Samples of 50 mg derived from decellularized, and native hearts were used to quantify the remaining genomic DNA. For this step, Illustra^®^ Tissue and Cells Genomic Prep Mini Spin Kit (GE Healthcare) was used in accordance with the manufacturer’s recommendations. The samples were digested with proteinase K and kit lysis buffer at 56°C for 2 h. The purified DNA samples from native and decellularized tissues were analyzed using a 260 nm spectrophotometer (Nanodrop, Thermo Scientific).

### 2.6. Immunofluorescence

Immunofluorescence for collagen type I (primary antibody anti-collagen I antibody (#600-401-103, 1:00, Rockland-Limerick, US) and elastin (primary antibody anti-elastin Abcam ab9519/Mouse Polyclonal/1:100) was performed in decellularized heart samples. While immunofluorescence for vimentin (primary antibody anti-vimentin Genetex GTX 35160/Mouse Polyclonal/1:100) and laminin (primary antibody anti-laminin alpha-2 #bs-8561R, 1:100, Rockland-Limerick, US) was performed in recellularized heart samples. For comparative purposes, these techniques were also performed on native heart samples. Immunofluorescence analysis for DAPI was performed for decellularized, recellularized, and native heart samples.

Native, decellularized, and recellularized samples were frozen and include in Tissue-Tek® OCT medium (#4583-1, Sakura Torrance, USA) at −150°C. Sections of 12 mm were cut in a cryostat (CM1520, Leica). The slides were fixed in 4% buffered paraformaldehyde (PFA) during 10 min, dried at room temperature (5 min), and incubated in PBS 1x + 10% goat serum for 1 h, and finally incubated overnight with primary anti-collagen I antibody, anti-elastin, anti-vimentin, and anti-laminin, in a humid chamber at 4°C. Subsequently, the slides were washed 3x during 5 min. After the slides were washed with PBS 1x + 1% goat serum at room temperature, incubated with a secondary antibody (Alexa Fluor^®^) for 1 h. The slides were chased in PBS 1x (3x/5 min), incubated with DAPI (1:10.000, Sigma-Aldrich) for 10 min at room temperature, and washed again with PBS 1x (3x for 5 min).

Furthermore, immunofluorescence was performed for DAPI (Invitrogen), and nuclei were stained with DAPI before mounting on slides using Shandon™ Immu-Mount (Thermo Fischer Scientific, Waltham, USA).

All slides were mounted and analyzed in a confocal microscope (FV1000 Olympus IX91, Japan) with 200× and 400× objectives, in five different microscopic fields, on a Nikon Eclipse E-80i fluorescence light microscope, from the Advanced Center for Diagnostic Imaging – CADI-FMVZ-USP.

### 2.7. Scaffold recellularization

For the scaffold recellularization process, equine skeletal muscle fibroblasts available on our cell repository (CEUA No. 4318311018) were used. This choice of cells was made for convenience since these fibroblasts were easily accessible and the recellularization step was performed only to assess the cell adhesion capacity of the produced scaffold.

The equine fibroblasts were incubated on 1 × 1 cm decellularized LV fragments in non-adherent plates, cultured in the cell growth medium at 37°C, 5% CO_2_ for 72 h. Samples from days 4, 6, and 8 underwent analysis with scanning electron microscopy (SEM) and immunofluorescence for vimentin and elastin as previously mentioned. The presence of cells was investigated by DAPI analysis.

### 2.8. SEM

This step was performed at the *Centro Avançado de Diagnóstico por Imagem* (Advanced Center for Imaging Diagnostics) at the *Faculdade de Medicina Veterinária e Zootecnia da Universidade de São Paulo (CADI-FMVZ-USP)*.

To observe collagen fibrils and ECM architecture, the samples were submitted on SEM analysis. Fragments from the native heart, decellularized and recellularized scaffolds were fixed in Karnovsky solution (v/v 1:1) for 48 h and 3 times washed with distilled water in an ultrasound bath for 5 min per wash. The fragments were post-fixed in 1% osmium tetroxide (SEM – Hatfield, USA) for 90 min, then dehydrated in an increasing series of ethanol (70–100%) under vigorous agitation. After this process, the sample was dried using an automated critical point dryer (ECM CPD300, Leica), transferred to stubs, and metalized with gold in a #K550, Emmitech (Ashford, United Kingdom) machine. The samples were analyzed and photographed using a Leo 435 VP scanning electron microscope (CADI/FMVZ).

## 3. Results

### 3.1. Macroscopic gross analysis after decellularization

**Figure 1:**
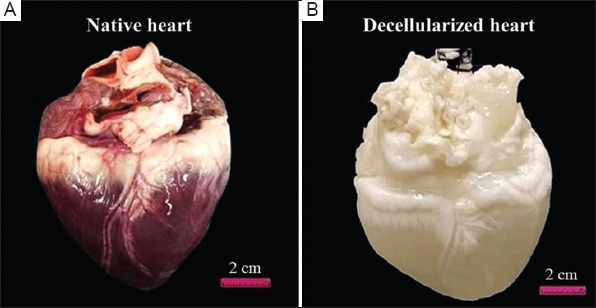
Macroscopic images. (A) Native porcine heart. (B) Decellularized porcine heart, showed that the heart was decellularized, with a greater volume left ventricular retained.

Macroscopic assessment showed marked differences between native and decellularized hearts. On the macroscopic analysis, the native heart presented usual conformation, reddish color in its body and apex region, as well as a presence of adipose tissue in the cardiac base region, right and left auricles of usual appearance, with vascular and arterial architecture preserved (Figure 1A).

**Figure 2:**
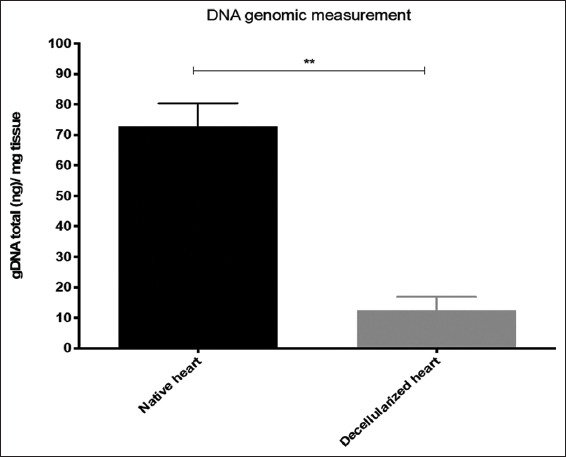
DNA measurement in native and decellularized heart samples. T tests analysis, with significant differences for **p≤0.05. Values expressed as mean and standard deviation.

The decellularized heart underwent significant morphological changes, especially regarding its color, assuming a whitish and translucent aspect. The cardiac architecture was preserved, as well the vessels walls and arteries from the heart base. On the epicardial surface, it was observed discrete linear structures, with a translucent appearance, corresponding to the space of the coronary arteries and veins (Figure 2A).

### 3.2. Quantification of remaining genomic DNA

In addition to macroscopic analysis described in Figure 1, the measurement of remaining genomic DNA showed values of 70 ng/mg for native cardiac, while in the decellularized heart, the value measured <20 ng/mg of tissue as recorded (Figure 2).

### 3.3. Histological analysis

**Figure 3:**
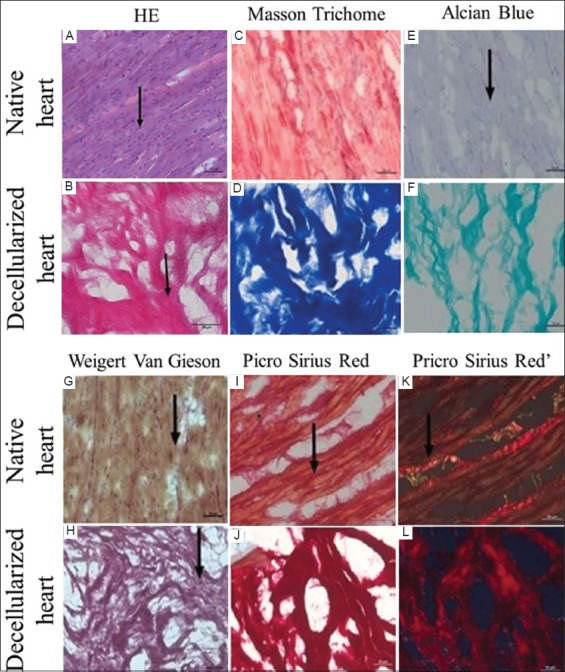
Microscopic analysis of native and decellularized porcine hearts. (A) Native heart – Cardiomyocyte nucleus shown in an arrow, stained by hematoxylin and eosin; (B) Decellularized heart – collagen shown in an arrow, stained by hematoxylin and eosin; (C) Native heart – staining of collagen fibers (dark red) and cardiomyocytes (reddish-pink), stained by Masson’s trichrome; (D) Decellularized heart – collagen fibers highlighted in blue stained by Masson’s trichrome; (E) Native heart – discrete tissue staining, arrow showing discrete area of GAGs, stained by Alcian Blue; (F) Decellularized heart – areas with evidenced GAGs highlighted in greenish-blue, stained by Alcian Blue; (G) Native heart – arrow pointing to a discrete area highlighted in dark red, corresponding to the collagen fiber, interspersed with cardiomyocytes, stained by Weigert-Van Gieson; (H) Decellularized heart – collagen fibers evidenced in dark red, stained by Weigert-Van Gieson; (I) Native heart – collagen fiber highlighted in dark red indicated by the arrow, peripheral to cardiomyocytes, stained with Picrosirius red; (J) Decellularized heart – collagen fibers intensely stained in dark red, stained by Picrosirius red; (K) Native heart – polarized light microscopy highlighting collagen fibers in bright red, stained by Picrosirius red; (L) Decellularized heart – polarized light microscopy with intense highlighting of collagen fibers in pale red, stained by Picrosirius red.

On tissues from the native heart cardiomyocytes, cells were arranged longitudinally, with its preserved fusiform or oval nuclei under HE staining. Furthermore, the striated muscle fibers were arranged transversally to the cardiomyocytes, in addition to Purkinje fibers. In cross-sections, endocardium, myocardium, and serous pericardium were organized and properly preserved (Figure 3A). On the samples from decellularized hearts stained by HE, cardiomyocyte nuclei were few observed, along with the presence of pink stained ECM, with spacing between collagen fibers, which were preserved (Figure 3B)

On the Masson’s Trichrome stained samples from the native heart, it was observed distinct reddish to pink staining of cardiomyocytes and collagen fibers (Figure 3C). On slides with decellularized heart tissue, Masson’s Trichrome stain showed the intense presence of collagen stained in blue (Figure 3D).

Slides from the native heart were lightly stained by Alcian Blue (Figure 3E), while it was possible to observe in the decellularized heart several organized areas stained in blue, corresponding to glycosaminoglycans (GAGs) (Figure 3F).

In native heart samples stained by Weigert-Van Gieson, it was observed that collagen fibers were stained in red and interspersed with cardiomyocytes stained in brown to yellow (Figure 3G). On the decellularized heart samples, the collagen fibers were intensely highlighted in dark red, presenting distinct spacing between them (Figure 3H).

On the native samples stained by Picrosirius red, a slight contrast in color between cardiomyocytes and collagen fibers was observed, assuming a darker hue in relation to these cells (Figure 3I); and lighter hue when observed under a polarized light microscope (Figure 3J). However, the collagen fibers were intensely stained on dark red and little hue differentiation was observed in the decellularized heart (Figure 3K). Under a polarized light microscope, these fibers were highlighted in light red, showing between them (Figure 3L).

### 3.4. Immunofluorescence analysis of decellularized heart

**Figure 4:**
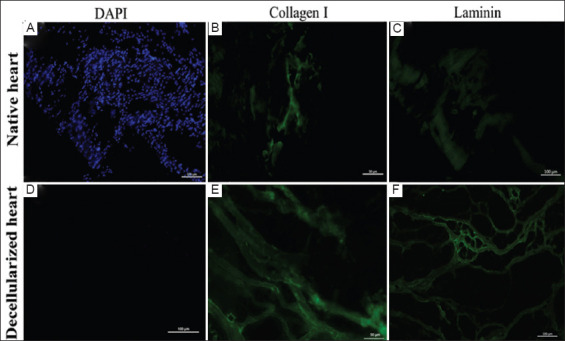
Immunofluorescence images from porcine sample hearts. (A) Native heart – positive immunofluorescence for DAPI; (B) Native heart – positive immunofluorescence for collagen type I; (C) Native heart – positive immunofluorescence for elastin; (D) Decellularized heart – negative immunofluorescence for DAPI; (E) Decellularized heart – positive immunofluorescence for collagen type I; (F) Decellularized heart – positive immunofluorescence for elastin.

Immunofluorescence for collagen type I and elastin labeling was performed for both native and decellularized heart. On the analysis obtained from images from the native heart, it was possible to observe positive marking for DAPI in blue (Figure 4A), positive marking in green for collagen type I (Figure 4B), and positive marking in green for elastin (Figure 4C). As for the immunofluorescence analysis of the decellularized heart, there was no labeling for DAPI, with negative results (Figure 4D), while for collagen type I, there was positive labeling in green, with detailed collagen fibers architecture (Figure 4E), and elastin was also marked in green (Figure 4F).

### 3.5. Recellularization analysis by immunofluorescence

**Figure 5:**
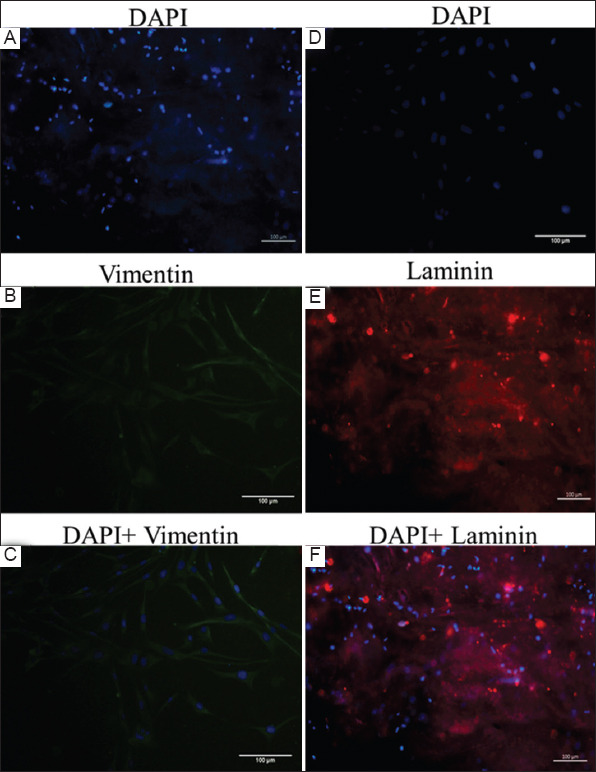
Immunofluorescence of recellularized scaffold. (A) Positive immunofluorescence for DAPI; (B) Positive immunofluorescence for vimentin; (C) Merge for DAPI+Vimentin immunostaining; (D) Positive immunofluorescence for DAPI; (E) Positive immunofluorescence for laminin; (F) Merge for DAPI+Laminin immunostaining.

The recellularization process was observed 48 and 72 h after the incubation of the cells on the scaffold. Fluorescence to nuclei marked by DAPI, immunofluorescence to vimentin and laminin were performed. Recellularized staining samples were positive for DAPI (blue cell nuclei), vimentin (green), and laminin (red) (Figure 5).

### 3.6. Scanning Electron Microscopy analysis

**Figure 6:**
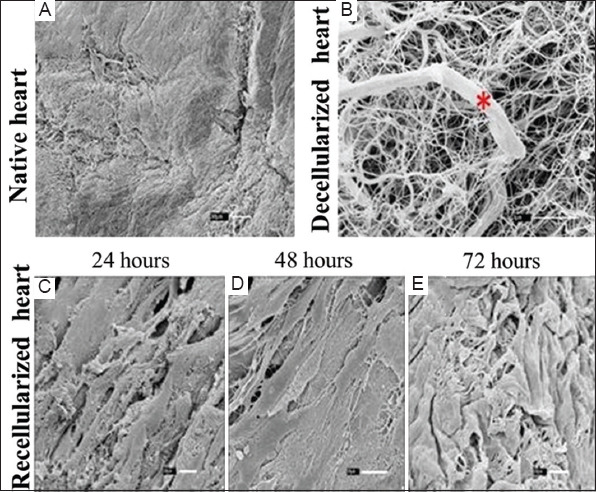
Scanning electron microscopy images. (A) Native heart showing the presence of cells and tissue integrity (10 μm); (B) Decellularized scaffold showing absence of cells and presence of extracellular matrix (5 μm); (C) SEM image from decellularized scaffold by equine muscle fibroblasts 24 h after incubation (10 μm); (D) SEM image from decellularized scaffold by equine muscle fibroblasts 48 h after incubation (10 μm); (E) SEM image from decellularized scaffold by equine muscle fibroblasts 72 h after incubation, with cells well adhered at its surface (10 μm).

Native heart samples were analyzed by SEM and sowed cells superficially grouped with the MEC between them (Figure 6A). Decellularized heart showed only ECM structure, with fibers present as well other structures like a blood vessel (*); while few cells were visualized on these samples (Figure 6B).

On the SEM analysis of the recellularized scaffold, the fibroblasts showed interaction with the ECM scaffold 24 h after its incubation; at this point, the image showed a monolayer of cells adhered to the scaffolds surface (Figure 5C). Other images were obtained 48 h (Figure 5D) and 72 h (Figure 5E) after fibroblasts incubation. During these periods, the surface of the sample was completely covered by cells, intermingled with the ECM demonstrating a firm cell adherence to scaffold.

## 4. Discussion

Organ and tissue decellularization have been proposed by several studies in bioengineering and resulting on functional mice hearts through cell remotion of native hearts [16], as well as the production of recellularized scaffolds from mice hearts using human Y1 induced pluripotent cells (iPSCs) [5].

A protocol for recellularization of lungs and heart of porcine was already described [10]. Recently, other studies established protocols to obtain ECM from hearts of porcines through hydrogels usage. Other studies described an automation of the pressure control that improved the whole porcine heart decellularization [8].

The present work described a protocol for decellularization of a whole porcine heart based on the use of SDS and Triton X-100 detergents through a perfusion system. After this procedure, color and organ shape changes were observed, assuming a whitish color and a translucent appearance, indicating the organ decellularization. The conical heart shape and architecture of cardiac vessels and arteries were preserved.

Some detergents were effective in the removal of cellular material from tissue due its ability to solubilize the cell membrane and dissociate proteins from DNA [2]. Porcine hearts have already been decellularized by means and protocols associating 3% Triton X-100 and 4% SDC, promoting a 92.2% DNA removal in 8 h [17]. The same method was applied again by gradually increasing the perfusion flow in the organ by the reagents [18]. In addition, authors mentioned that the addition of SDS or Triton X-100 to decellularization protocols can improve this process [19]. However, these agents can influence in protein structure disruption and loss of matrix components, especially collagen and GAGs [20].

The use of SDS showed to be adequate to remove cellular components from the ECM, reducing inflammatory reactions and rejections in later transplants; however, some studies observed that SDS also had the potential to remove parts of the ECM components [21].

Regarding the impact of detergent-based decellularization methods on porcine tissues for heart valve engineering, both SDS and Triton X-100 had a similar effect on the ECM; additionally, these decellularization agents had more impact on myocardium and pericardial tissue than on heart valve leaflets [9,13].

The DNA measurement performed in this work showed decellularized samples presenting a considerable absence of cellular material and probably losing immunogenic factors as well, when compared to the native sample organ. Considering the quality parameters for decellularization, the ECM must not contain more than 50 ng of DNA/mg of dry weight, DNA fragments cannot exceed 200 bp, and no nuclear components can be visible [2]. In this study, the amount of remaining genomic DNA in the decellularized sample prevailed within the expected parameter presenting <20 ng of DNA/mg of dry weight.

The visualization of few nuclei in the decellularized sample slides stained by HE indicates that there was an intense decellularization of cardiomyocytes. It is important to reinforce the presence of GAGs in decellularized cardiac laminae stained by Alcian Blue, due to its critical role in the interaction of the biological process, such as regulation of signaling molecule activity, cell proliferation, control of cell molecule traffic, co-receptors, adhesion and cell migration, basement membrane selectivity, and immune response [22]. The histological analysis suggested that decellularization protocol established in this study preserved the collagen fibers.

Through immunofluorescence analysis, we found that collagen type I and elastin were present, but the cell (negative DAPI) in the decellularized scaffolds was absent, indicating the success of the protocol developed. The presence of collagen and elastin fibers in the ECM of decellularized samples are essential to the mechanical properties and elasticity of the scaffold, since these proteins act as signaling molecules for cell differentiation and proliferation in addition to maintain three-dimensional ECM structure [22]. The negative results for DAPI also indicate the absence of cells nuclei, as observed on histological analysis.

There was good adhesion and cell proliferation in the recellularization process, which indicates that the cardiac framework obtained from the protocol developed in the present study was able to preserve the biological interaction between the ECM and the cells [23]. These results are supported by immunofluorescence analyses that was positive for vimentin and laminin proteins and DAPI labeling [24]. Furthermore, the association of equine fibroblast cells with the ECM scaffold was observed in the SEM analysis. There was complete recellularization after 8 h of cell incubation; and based on these results and on the parameters established by literature [2], it was possible to state that the established protocol can be applied in tissue and organ engineering in the future. The porcine heart scaffold can be used in the recellularization processes and it is an important subject to future applications in tissue engineering or regenerative medicine.

Based on mentioned qualitative analysis, this study provided a good experimental and an alternative low-cost protocol to decellularize the whole porcine heart; optimizing the time and reducing the use of detergents, since the process of decellularization aims to keep ECM components without damaging or losing tissue after its appliance [20].

## 5. Conclusion

The present study developed an efficient and low-cost experimental protocol for porcine heart decellularization that can be used in recellularization process. According to the results obtained in this work, both macroscopically and microscopically, the established protocol minimized ECM loss. Is possible to state that the decellularization protocol described has a promising approach to tissue bioengineering, preserving the ECM and the porcine heart architecture.

## Figures and Tables

**Supplementary Figure 1:**
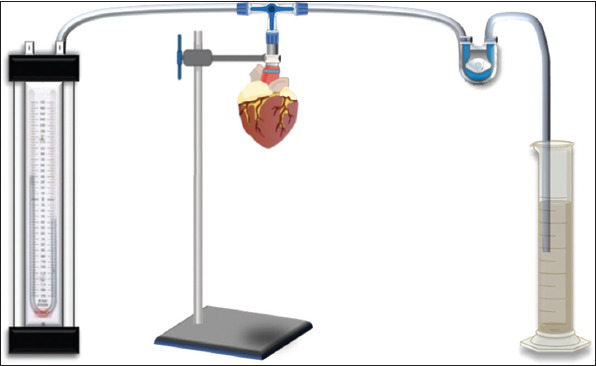
Experimental design for organ compliance analysis

**Supplementary Figure 2:**
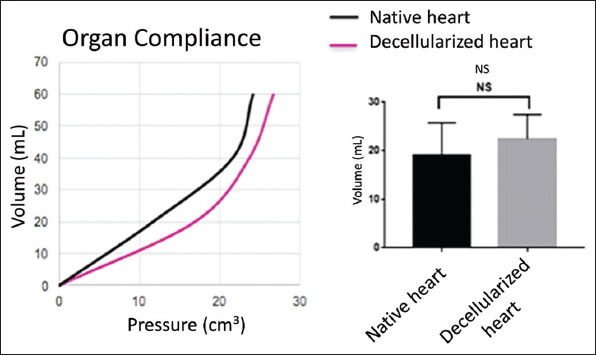
Complacency. Relation of the volume injected into the organ and the pressure exerted on it, both in the heart before and after being decellularized (scatter plot). Statistical analysis *t-*test did not show significant differences between them.

**Supplementary Figure 3:**
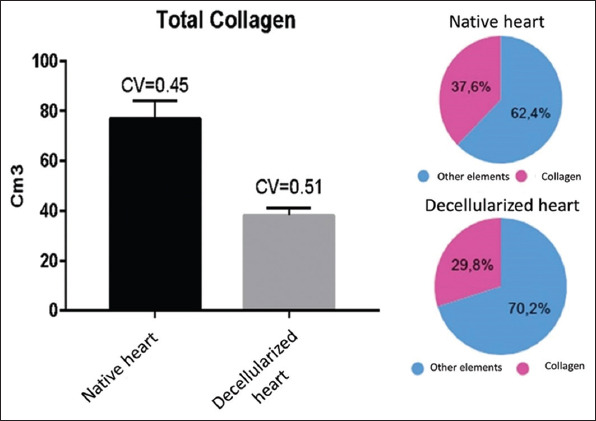
Total collagen. Density in percentage volume of total collagen in native heart and decellularized heart

**Supplementary Figure 4:**
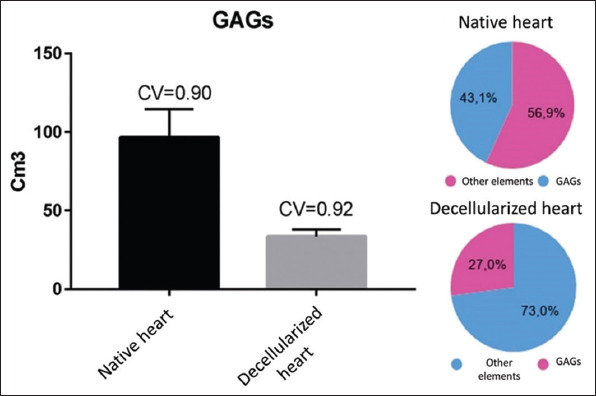
Total GAGs density in percentage volume of GAGs in native heart and decellularized heart

## Appendix

**ORIGINAL ARTICLE**

Supplementary data: Development of a new decellularization protocol for the whole porcine heart

**1. Material and Methods**

*1.1. Material, reagents, and decellularization protocol*

This study was authorized by the Animal Experimentation Ethics Committee (1166080618). Adult pig hearts of both sexes obtained under clean conditions from a slaughterhouse were used, each heart weighing approximately 300 g. These hearts were submitted to decellularization adapted based on the previously literature [1-6], and the one with best performance is described below and detailed in this work.

For detergents perfusion, the aorta was cannulated with a silicon tube. Then, the heart was placed in a beaker containing 4 L of 0.1% phosphate-buffered saline solution (PBS), in a circulating system of 400 mL/min for 30 min, performed by a peristaltic pump (Harvard Peristaltic Pump – Harvard Apparatus). After this procedure, Milli-Q water was perfused in the same system. Four liters of 4% SDS were perfused through myocardial circulation, at 400 mL/min for 24 h. This process was repeated for 6 days, and then, 1% Triton X-100 was infused followed by PBS 0.1% solution and Milli-Q water for 24 h to remove cell debris and chemical residues in the sample.

*1.2. Organ compliance and left ventricle (LV) volume estimation*

Heart complience was performed before and after the decellularization process. The heart was coupled in an experimental system (Supplementary Figure 1) that contains a manometer, beaker, and a peristaltic pump, connected together. For every 20 ml of water injected into the organ, the pressure was measured and documented for later interpretation.

To estimate the volume of the LV of the decellularized heart, Cavalieri’s Principle [7] was applied, applying the following the formulas bellow:

V(P) = ∑pp x a(p) x t

V(L) = ∑pl x a(p) x t

Where;

V(P): Left ventricular wall volume

∑pp: Sum of points that touched the LV wall

a(p): Area per point: 0.4 cm (for LV wall) t: Cut

thickness: 2 cm

V(L): Lumen volume

∑pl: Sum of the points that touched the lumen a(p):

Area per point: 0.25 cm (for LV lumen)

The LV volume was obtained by adding V (P) and V (L). The error coefficient of the Cavalieri’s Principle was estimated according to Gundersen *et al*. (1999).

*1.3. Collagen and glycosaminoglycans (GAGs) quantification*

To estimate the total volume of collagen and GAGs of extracellular matrix (ECM), a point system was used that was superimposed on the histological sections sampled following the SURS pattern, where the fraction of volumes was calculated from the formula bellow:

Vv(col) = ∑Pcol/∑Pframe

Where:

∑pcol: Sum of points that touched the collagen area characterized by Picrosirius staining and GAGs by Alcian Blue staining

∑frame: Sum of points that touched the region of intensity (VE)

**2. Results and Discussion**

*2.1. Organ compliance and LV volume estimation*

Regarding the estimate of LV total volume, the decellularized heart obtained 90.5 cm³, with 29% of volume retraction compared to its initial volume, before decellularization process (Supplementary Figure 2). In this experiment, organ compliance was assessed before and after the decellularization process, where no significant differences were observed between both native and decellularized hearts, reinforcing the idea of an ideal scaffold for future applications (Supplementary Figure 2).

Ferng *et al*. in their studies developing a new protocol for decellularization of a whole porcine heart also reported a reduction in heart weight after decellularization [8]. However, this did not compromise the subsequent successful recellularization of these organs. Furthermore, despite the observed retraction, good results were obtained from microscopic and recellularization analysis in our study.

Regarding the organ compliance, Momtahan *et al*. performed a test of mechanical properties of the heart; decellularized heart samples compared to native hearts had their volume calculated, and then, the compression was measured by stress as a function of the deformation of these samples, to assess their mechanical stiffness [1]. They concluded that decellularized samples tend to have a smaller elasticity and resistance to compression stress than native tissue samples. The findings of our work indicate that the decellularized heart had a positive characteristic regarding resistance to stress due to dilation.

*2.2. Collagen and GAGs quantification analysis*

To quantify the total volume of collagen and GAGs, a *t*-test for control and decellularized hearts was used. The variation coefficient was <1, giving credibility to the sampling.

The stereological analysis carried out in the present study showed that the total collagen present in the native cardiac tissue (control) made up 38% of the total volume of the LV. In the decellularized heart, collagen reached 30%, suggesting that the decellularization process used preserved collagen fibers, even during/after successive washes with SDS, distilled water, and PBS. It is also interfered that this would be a good biomaterial for recellularization, since it kept a large part of its three-dimensional structure (Supplementary Figure 3).

As for GAGs, their total volume in the LV control reached 43%, while the decellularized heart reached 27% (Supplementary Figure 4) – important information, in view of the important role of GAGs in interactions in biological processes, such as the regulation of the activity of signaling molecules, cell proliferation, control of cell and molecule traffic, coreceptors, cell adhesion and migration, basal membrane selectivity, and immune response [9].

**3. Conclusion**

The supplementary data inserted in this file strengthen the quality of the scaffold obtained from the new decellularization protocol described in this work, since a good organ compliance and preservation of collagen and GAGs were also observed.

**References**

[1] Momtahan N, Poornejad N, Struk JA, Castleton AA, Herrod BJ, Vance BR, *et al*. Automation of Pressure Control Improves Whole 9 Porcine Heart Decellularization. Tissue Eng Part C Methods 2015;21:1148-61.

[2] Roosens A, Somers P, de Somer F, Carriel V, van Nooten G, Cornelissen R. Impact of Detergent-Based Decellularization Methods on Porcine Tissues for Heart Valve Engineering. Ann Biomed Eng 2016;44:2827-39.

[3] Ye X, Wang H, Gong W, Li S, Li H, Wang Z, *et al*. Impact of Decellularization on Porcine Myocardium as Scaffold for Tissue Engineered Heart Tissue. J Mater Sci Mater Med 2016;27:70.

[4] Haupt J, Lutter G, Gorb SN, Simionescu DT, Frank D, Seiler J, *et al*. Detergent-Based Decellularization Strategy Preserves Macro-and Microstructure of Heart Valves. Interact Cardiovasc Thorac Surg 2018;26:230-6.

[5] Sakina R, Llucià-Valldeperas A, Lourenço AH, Harichandan A, Gelsomino S, Wieringa P, *et al*. Decellularization of Porcine Heart Tissue to Obtain Extracellular Matrix Based on Hydrogels. Methods Cell Biol 2020;157:3-21.

[6] Godehardt AW, Tönjes RR. Xenotransplantation of Decellularized Pig Heart Valves-Regulatory Aspects in Europe. Xenotransplantation 2020;27:e12609.

[7] Gundersen HJ, Jensen EB, Kiêu K, Nielsen J. The Efficiency of Systematic Sampling in Stereology-Reconsidered. J Microsc 1999;193:199-211.

[8] Ferng AS, Connell AM, Marsh KM, Qu N, Medina AO, Bajaj N, *et al*. Acellular Porcine Heart Matrices: Whole Organ Decellularization with 3D-Bioscaffold and Vascular Preservation. J Clin Transl Res 2017;3:260-70.

[9] Heath DE. A Review of Decellularized Extracellular Matrix Biomaterials for Regenerative 4 Engineering Applications. Regen Eng Transl Med 2019;55:155-66.
